# Editorial: Experimental Manipulations to Predict Future Plant Phenology

**DOI:** 10.3389/fpls.2020.637156

**Published:** 2021-01-14

**Authors:** Janet S. Prevéy, Yann Vitasse, Yongshuo Fu

**Affiliations:** ^1^U.S. Geological Survey, Fort Collins Science Center, Fort Collins, CO, United States; ^2^Swiss Federal Institute for Forest, Snow and Landscape Research, Forest Dynamics, Birmensdorf, Switzerland; ^3^Beijing Normal University, Beijing, China

**Keywords:** plant phenology, climate change, vegetation, mechanistic understanding, photoperiod, temperature, warming

Plant phenology is one of the most visible biological indicators of ongoing climate change. Shifting phenological events, such as earlier leaf-out and flowering in spring and overall delayed leaf senescence in fall, have been observed around the world over the past few decades, and these changes have the potential to substantially affect plant fitness and distribution (Chuine, 2010), plant-animal interactions (Rudolf, 2019), as well as regional and global-scale carbon, water, and energy balances (Richardson et al., 2013). However, how plant phenology will continue to shift with future climate change remains uncertain. For example, as temperatures rise, plants may not experience winter conditions that are cold enough to satisfy a complete dormancy release, making leaf-out less sensitive to warmer temperatures in spring (Fu et al., 2015). For summer and fall phenophases, drought and hot spells may advance or delay leaf senescence and can alter nutrient reallocation. In order to adequately predict the future dynamics of vegetation–climate systems, it is therefore essential to understand the driving factors of plant phenology and how they may change under a warmer climate. To date, most of the published literature consists of observational studies of plant phenological responses to environmental variation over time, and models are generally calibrated using long-term series of observations. However, future predictions require running simulations beyond the range of historical conditions, and seasonal changes in temperature, photoperiod, and soil moisture are often highly correlated (Flynn and Wolkovich, 2018; Ettinger et al., 2020). This further complicates the acquisition of accurate parameters that reflect the physiological functioning of plant development in response to environmental cues. Experimental studies are therefore key and urgently needed to isolate the different drivers and to examine how they separately and interactively influence changes in plant phenology.

The articles presented here address several of the outstanding gaps in knowledge of plant phenology using experimental manipulations. These studies were conducted in a wide range of ecosystems, from the coldest locations on the planet in Arctic tundra, to ecosystems with extreme seasonal fluctuations on the Tibetan Plateau, to some of the driest regions in semi-arid deserts. These studies also utilized a diversity of manipulative methods to examine plant responses to environmental cues, including passive open-top chambers, active electrical heaters, climate-controlled growth chambers, as well as making use of natural climatic and micro-climatic gradients as experimental treatments to test phenological differences.

Four of the manuscripts in this Research Topic used manipulative experiments to parse the effects of potential environmental and biological drivers on plant phenology by isolating these driving variables, while controlling for others, in climate-controlled growing chambers or open-top warming chambers. Wang et al. placed twigs of six shrub and tree species in growing chambers to examine the interactive effects of chilling, forcing, and photoperiod on the timing of flowering. They found that increases in chilling and forcing temperatures advanced phenology for most of the species, while longer daylength significantly advanced phenology for only two of the species, especially under low chilling conditions. Malyshev et al. Similarly, found that experimental warm spells advanced leaf-out timing of European beech and birch seedlings when applied after substantial exposure to chilling temperatures, whereas warm spells deepened dormancy and therefore delayed leaf-out when they occurred in fall for European beech. Hu et al. found that increasing winter temperature with passive warming using open-top chambers in an alpine meadow on the Tibetan Plateau advanced the timing of flowering and fruiting for most plant species. Here again, changes in the timing of reproductive phenology varied by plant species. Not only abiotic drivers influence plant phenology; phenological shifts might also be influenced by plant-animal interactions. Ulrich et al. used innovative climate-controlled chambers in the iDiv Ecotron experimental setup to test effects of invertebrate density on plant phenology. They found that a greater density of invertebrates advanced the phenology of one plant species but delayed the phenology of another, in what might be one of the first studies to experimentally examine the effects of a biotic driver on changes in plant phenology.

Three studies in this collection illustrate how plant compositional changes, in combination with experimental manipulations, ultimately influence community-level shifts in phenology. In a long-term, large-scale warming experiment conducted across eight tundra sites, May et al. found that warming shifted the length of the growing season in some sites and not others, and these differences depended on plant species composition and growth forms at each site. In another high Arctic tundra location, Chisholm et al. examined plant phenology along topographical variations in permafrost melt and observed that phenology can be delayed in permafrost depressions compared to stable ground. These changes are due to both shifts in plant species composition to later-blooming species in permafrost depressions, as well as to later timing of phenological events within species. Meng et al. conducted a reciprocal transplant study in the Tibetan plateau and found that the temperature sensitivity of community-level flowering was strongly influenced by changes in the composition of flowering functional groups in transplanted plots, as well as by direct responses to changes in temperature along the climatic gradient.

To date, there have been few experimental studies of plant phenology in desert ecosystems. Here, Howell et al. and Bao et al. examine the experimental effects of warmer temperatures and changes in precipitation on plant phenology in desert ecosystems in North America and China. Howell et al. found that warmer temperatures advanced the timing of flowering and senescence of the early-growing invasive grass *Bromus tectorum*, and led to an overall shortening of the growing season for the grass. Bao et al. found that water addition treatments advanced leaf-out and delayed leaf senescence of the dominant shrub *Nitraria tangutorum*, indicating that increased precipitation may lengthen the growing season of this shrub in desert ecosystems.

The final three studies highlight innovative methods to measure plant phenological changes in response to historical and simulated climate change. Davis et al. describe and test a machine-learning method that accurately identifies buds, flowers, and fruits on digitized herbarium specimens, and can thus allow for large-scale phenological analyses of vast natural history collections increasingly available online. Menzel et al. illustrate a simple methodology and introduce an easy-to-use Shiny application (available at www.baysics.de) to measure how chilling and forcing temperatures influence phenology of cut twigs that can be implemented by citizen scientists and students. Finally, Frei et al. test how open-top chambers in combination with active heating can accurately simulate temperature changes for larger plants and trees. This quantitative assessment indicates that warming experiments involving taller vegetation should incorporate both passive and active warming to achieve consistent temperature differences between treatments.

Results from this collection of articles show that phenological responses can vary greatly among species, ecosystems, and even microsites. This suggests that future plant phenological changes in response to climate change may be more nuanced than generalized advances of phenological events per degree temperature increase across entire vegetation types. Identifying phenological responses to environmental drivers through manipulative experiments is crucial for a comprehensive understanding of the response of species and ecosystems to ongoing climate change and for better predictions of future phenological shifts.

## Author Contributions

JP drafted the first version of the editorial. YV and YF made extensive edits, additions, and revisions. All authors contributed to the article and approved the submitted version.

## Conflict of Interest

The authors declare that the research was conducted in the absence of any commercial or financial relationships that could be construed as a potential conflict of interest.
